# Tailoring the Thermal and Mechanical Properties of PolyActive^TM^ Poly(Ether-Ester) Multiblock Copolymers Via Blending with CO_2_-Phylic Ionic Liquid

**DOI:** 10.3390/polym12040890

**Published:** 2020-04-12

**Authors:** Martina Klepić, Alessio Fuoco, Marcello Monteleone, Elisa Esposito, Karel Friess, Zuzana Petrusová, Pavel Izák, Johannes Carolus Jansen

**Affiliations:** 1 Department of Physical Chemistry, University of Chemistry and Technology Prague, Technická 5, 166 28 Prague 6, Czech Republic; mklepic0@gmail.com (M.K.); karel.friess@vscht.cz (K.F.); izak@icpf.cas.cz (P.I.); 2 Institute on Membrane Technology (CNR-ITM), Via P. Bucci, 17/C, 87036 Rende (CS), Italy; a.fuoco@itm.cnr.it (A.F.); m.monteleone@itm.cnr.it (M.M.); 3 Czech Academy of Sciences, Institute of Chemical Process Fundamentals, Rozvojová 135, 165 02 Prague 6—Suchdol, Czech Republic; petrusova@icpf.cas.cz

**Keywords:** block copolymer membranes, ionic liquid, blends

## Abstract

The last decade has seen an exponential increase in the number of studies focused on novel applications for ionic liquids (ILs). Blends of polymers with ILs have been proposed for use in fuel cells, batteries, gas separation membranes, packaging, etc., each requiring a set of specific physico-chemical properties. In this work, blends of four grades of the poly(ether-ester) multiblock copolymer PolyActive™ with different concentrations of the CO_2_-philic 1-butyl-3-methylimidazolium bis(trifluoromethylsulfonyl)imide [BMIM][Tf_2_N] were prepared in the form of dense films by a solution casting and solvent evaporation method, in view of their potential use as gas separation membranes for CO_2_ capture. Depending on the polymer structure, the material properties could be tailored over a wide range by means of the IL content. All samples were dry-feeling, highly elastic self-standing dense films. The microstructure of the blends was studied by scanning electron microscopy with a backscattering detector, able to observe anisotropy in the sample, while a special topographic analysis mode allowed the visualization of surface roughness. Samples with the longest poly(ethylene oxide terephthalate) (PEOT) blocks were significantly more anisotropic than those with shorter blocks, and this heterogeneity increased with increasing IL content. DSC analysis revealed a significant decrease in the melting enthalpy and melting temperature of the crystalline PEOT domains with increasing IL content, forming an amorphous phase with *T*_g_ ≈ −50 °C, whereas the polybutylene terephthalate (PBT) phase was hardly affected. This indicates better compatibility of the IL with the polyether phase than the polyester phase. Young’s modulus was highest and most IL-dependent for the sample with the highest PEOT content and PEOT block length, due to its high crystallinity. Similarly, the sample with short PEOT blocks and high PBT content also showed a high modulus and tensile strength, but much lower maximum elongation. This study provides a detailed discussion on the correlation between the morphological, thermal, and mechanical properties of these PolyActive™/[BMIM][Tf_2_N] blends.

## 1. Introduction

Polyethene oxide terephthalate-polybutylene terephthalate (PEOT-PBT) multiblock copolymers are well-known semi-crystalline polymers commercialized under the name PolyActive™. Their application ranges from pharmaceutics [1,2] and medicine [3,4] to gas separation membranes [5,6]. Poly(ethylene oxide) (PEO)-based membranes have been recognized as promising materials for CO_2_ separation [7]. Polar ether oxygen groups in PEO interact favorably with CO_2_, resulting in its high solubility selectivity [8,9]. Bondar et al. [10] reported CO_2_/H_2_ selectivities of 9.8 and CO_2_/N_2_ selectivities of 56, with CO_2_ permeability coefficients of approximately 220 Barrer in polyether-*b*-polyamide segmented block copolymers. Car et al. [11], for the PEOT-PBT copolymer, reported a CO_2_ permeability of 115 Barrer and CO_2_/H_2_ selectivities of 10.2.

The morphologies of block copolymers are very complex and can contain up to five different phases depending on the composition, namely crystalline hard and soft segments, amorphous hard and soft segments, and an interfacial region between the hard and soft segments [12]. Thus, knowledge of the phase behavior is of crucial importance for the understanding of their mass-transport properties. Barbi et al. [13] obtained useful information on the structure-property relationships by small-angle X-ray scattering (SAXS) measurements on five commercial grades of polyether-block-polyamide copolymers (PEBA) with varying soft block fraction and varying chemical compositions. A more extensive structure-property study was made with PEO-PBT block copolymers by Metz et al. [14]. The authors addressed the effects of various amounts of hard phase with constant segment length; soft segment length variation with a constant soft-hard ratio; and effect of the melting temperature and degree of PEO crystallinity. They concluded that CO_2_-philic membranes should have a high soft-block content for higher solubility, and a high length but low crystallinity of the soft segment to elevate the chain flexibility and permeance. Many modifications and improvements to the PEO-containing copolymers have been made to achieve this.

The simplest approach to optimize CO_2_-selective membranes is to blend the copolymers with low-molecular-weight compounds. Studies on blends of low-molecular-weight poly(ethylene glycol) (PEG) with Pebax^®^ and PolyActive™ by Car et al. [11,15] reported that the use of PEG as a spacer decreased crystallinity in the membranes and improved the performance of pure copolymers. However, PEG only blended with the PEG phase of the copolymer, leaving the hard phase intact and lowering the number of free ethylene oxide (EO) units, thus giving only a modest increase in permeability. To overcome these limitations, PEG with different end groups [16] or PEG-functionalized POSS [17,18] and PDMS [19] were used. Incorporation of the PEG-dibutyl ether (PEG-DBE) into PEO-PBT limited the hydrogen bonding, and its moderate compatibility with PBT improved the CO_2_-philic character of this phase and increased the interchain distance, resulting in a five-fold increased CO_2_ permeability of membranes.

A PEBAX^®^1657 blend membrane containing 50 wt.% of a PDMS–PEG additive exhibited an increase in CO_2_ permeability from ∼100 to ∼530 Barrer. A 30 wt.% PEG-POSS-containing nanocomposite showed an improvement in gas permeability without any significant change in selectivity. However, blends of low-molecular-weight compounds and polymers have the limitation of being unstable in time, undergoing thermal transitions, and being subject to leaching-out of low-molecular-weight compounds [20].

Another concept to simultaneously improve the permeability and selectivity of the CO_2_ separation membranes is that of mixed matrix membranes (MMMs) [21,22]. Sabetghadam et al. [23] fabricated thin MMMs based on nanosheets of the Cu-BDC and PolyActive™ polymer, improving the CO_2_/N_2_ selectivity of the thin membranes up to 77 and the CO_2_ permeance to 40 GPU (1 GPU = 1.0 × 10^−6^ cm^3^(STP) cm^−2^ s^−1^ cmHg^−1^). Liu et al. [24] reported on a thin-film composite membrane (TFCM) fabricated by spin-coating of the ultrathin metal-organic framework (MOF) gutter layer with PolyActive^TM^ exhibiting a CO_2_ permeance of ∼2100 GPU with a CO_2_/N_2_ ideal selectivity ∼30.

Another class of tailorable materials is formed by ionic liquid membranes, usually offering a combination of high diffusivity and good selectivity. Dai et al. [25] prepared TFCM with Pebax^®^ and a task-specific ionic liquid (TSIL) blend selective layer and reported on a significant increase in CO_2_ permeance and CO_2_/N_2_ selectivity by incorporation of TSIL. The use of ionic liquids (ILs) to enhance polymer properties is a very promising and fast-growing research topic [26]. Mass transfer in ILs is faster than in polymeric membranes [27], their separation properties can be adjusted for specific applications by proper selection of the anion and cation, and they exhibit high solubility of CO_2_ compared to other gases [28]. Addition of 20 to 80 wt.% of 1-butyl-3-methylimidazolium trifluoromethane sulfonate, [BMIM][CF_3_SO_3_], to Pebax^®^1657 was reported to give a fourfold increase in CO_2_ permeability with a modest decrease in CO_2_/N_2_ selectivity from 60 to 40 [29]. Estahbanati et al. [30], for a blend of Pebax^®^1657 with 50 wt.% of 1-butyl-3-methylimidazolium tetrafluoroborate ([BMIM][BF_4_]), reported an increase in CO_2_ permeability from 110 to 190 Barrer and CO_2_/N_2_ selectivities from 78.6 to 105.6. Jansen et al. [31] prepared ionic liquid polymeric gel membranes containing 20 to 80 wt.% of the ionic liquid 1-ethyl-3-methylimidazolium bis(trifluoromethylsulfonyl)imide ([EMIM][Tf_2_N]) in poly(vinylidene fluoride-co-hexafluoropropylene) (p(VDF-HFP)). Increasing IL concentration resulted in the rise in gas permeability and CO_2_/H_2_ selectivity and data exceeding the general trend in the Robeson diagram of the CO_2_/H_2_ selectivity against the CO_2_ permeability. Such promising results show that ILs present a very perspective additive for copolymers to create a CO_2_-selective membrane with improved properties.

**Figure 1:**
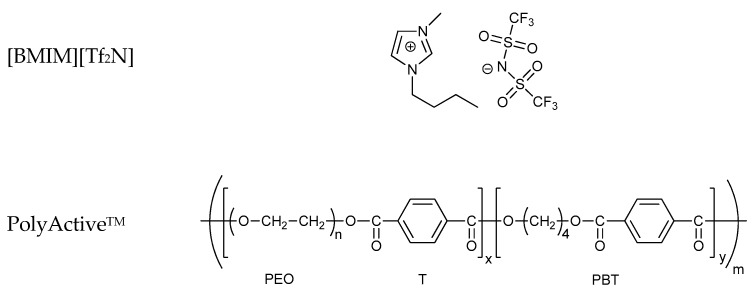
Molecular structure of 1-butyl-3-methylimidazolium bis(trifluoromethylsulfonyl)imide [BMIM][Tf_2_N] and generalized structure of PolyActive^TM^.

In this work, we prepared four series of blend membranes with 1-butyl-3-methylimidazolium bis(trifluoromethylsulfonyl)imide ([BMIM][Tf_2_N]) IL and PolyActive™, a block copolymer with interesting perspectives for CO_2_ separation (Figure 1). Thorough structural and morphological studies of the blend membranes were made by SEM observations, tensile tests, and DSC analysis. The effect of the IL content on the properties was examined for six membranes with different IL concentrations, while the effect of the PolyActive™ structure (with a different soft–hard segment ratio and different *M*_w_ of the soft segment) was evaluated by selecting four different grades of PolyActive™. The study aimed to determine structure-property relationships as a future guide for practical applications of the films in, for instance, membranes for gas or vapor separation. The main aim of this work was to identify and understand the relationships between the structure and properties of these polymeric materials, as a future guide for its wider material applications. Indeed, thorough knowledge of the properties of polymer films will allow the design and preparation of membranes with tailored properties, for example, for efficient separation of gases or gas/vapor mixtures.

## 2. Materials and Methods

Chloroform (99%) was purchased from Penta Chemicals (Prague, Czech Republic) and VWR (Milan, Italy) and was used as the solvent for membrane preparation. All gases for the permeation tests were supplied by Sapio (Monza, Italy) at a minimum purity of 99.9995+%. The ionic liquid used in this study was 1-butyl-3-methylimidazolium bis(trifluoromethylsulfonyl)imide (abbreviation [BMIM][Tf_2_N], ≥98%), and was purchased from Sigma-Aldrich (Prague, Czech Republic). Two grades (1500PEOT77PBT23 and 4000PEOT77PBT23) of the thermoplastic elastomer PolyActive^TM^, a polyethylene oxide terephthalate-polybutylene terephthalate (PEOT-PBT) multiblock copolymer, were purchased from PolyVation B.V., Groningen, The Netherlands, who supplied two other grades, 1000PEOT55PBT45 and 4000PEOT50PBT50, as a free sample. The first number in the code stands for the molecular weight of the PEO block, and the second and third numbers indicate the fractions (in wt.%) of the PEOT and the PBT blocks, respectively (Table 1). The generalized structure of PolyActive^TM^ PEOT-PBT copolymers and the chemical structure of [BMIM][Tf_2_N] are given in Figure 1.

### 2.1. Membrane Preparation

Polymer solutions of 5 wt.% of **P1**, **P3**, and **P4** grades of PolyActive^TM^ in chloroform were prepared at room temperature under stirring for 2 h. Different amounts of the ionic liquid [BMIM] [Tf_2_N] were added to the polymer solutions and stirred for another 2 h at room temperature. PolyActive^TM^ grade **P2** was dissolved at 3 wt.% in chloroform, by stirring for 2 h close to the boiling temperature (ca. 60 °C), yielding a slightly hazy solution. After adding the required amounts of ionic liquid, the solution was stirred for another 2 h at 60 °C. The final polymer/IL/chloroform solutions were poured into Teflon Petri dishes. The Petri dishes were partially covered to ensure slow evaporation of the solvent and to avoid contamination by dust particles. The solvent was evaporated at 35 °C for at least 24 h until the film was formed. **P2** samples showed shrinkage upon solvent evaporation. The films were then easily removed from the Petri dishes. Table 1 gives an overview of the sample characteristics.

### 2.2. Characterization

#### 2.2.1. DSC

DSC analysis was carried out using a Pyris Diamond Differential Scanning Calorimeter (Perkin Elmer, USA) equipped with an intracooler refrigeration system. Samples of 15–30 mg were packed in small baskets of household aluminum foil (6.8 mg). First, the samples were heated from room temperature to +250 °C and kept at this temperature for 1 min; then, the samples were cooled down to −75 °C where they were kept for 5 min and lastly heated up again to +250 °C. The DSC runs were performed at a scan rate of 15 °C min^−1^. Before the measurements, the samples were kept at 50 °C under vacuum for one night to remove adsorbed moisture. The pure ionic liquid was analyzed as a reference in the range from −100 to +200 °C, with fast cooling or annealing below the melting point to suppress or to enhance crystallization.

#### 2.2.2. SEM

The morphology of the membranes was studied using scanning electron microscopy (SEM, Phenom ProX Desktop model), applying an acceleration voltage of the primary electron beam of 5 kV. SEM images were acquired in backscatter electron imaging mode and topographic mode at a magnification of 1000× and 5000× without sputter coating with gold.

#### 2.2.3. Mechanical Tests

Tensile tests were carried out at room temperature on a Zwick/Roell single-column Universal Testing Machine, model Z2.5, equipped with a 50 N load cell and pneumatic clamps. The surface of one flat clamp was covered with adhesive rubber to avoid slipping or damage of the samples, while the second clamp had a rounded surface to avoid extraction of the sample. The average value and the standard deviation of Young’s modulus, the break strength, and the maximum deformation were determined on a series of at least three samples. The sample width was 5 mm, and the grip-to-grip distance was 40 mm. The deformation rate was 80 mm min^−1^ (200% min^−1^).

## 3. Results and Discussion

### 3.1. Membrane Morphology and Microstructure

**Figure 2:**
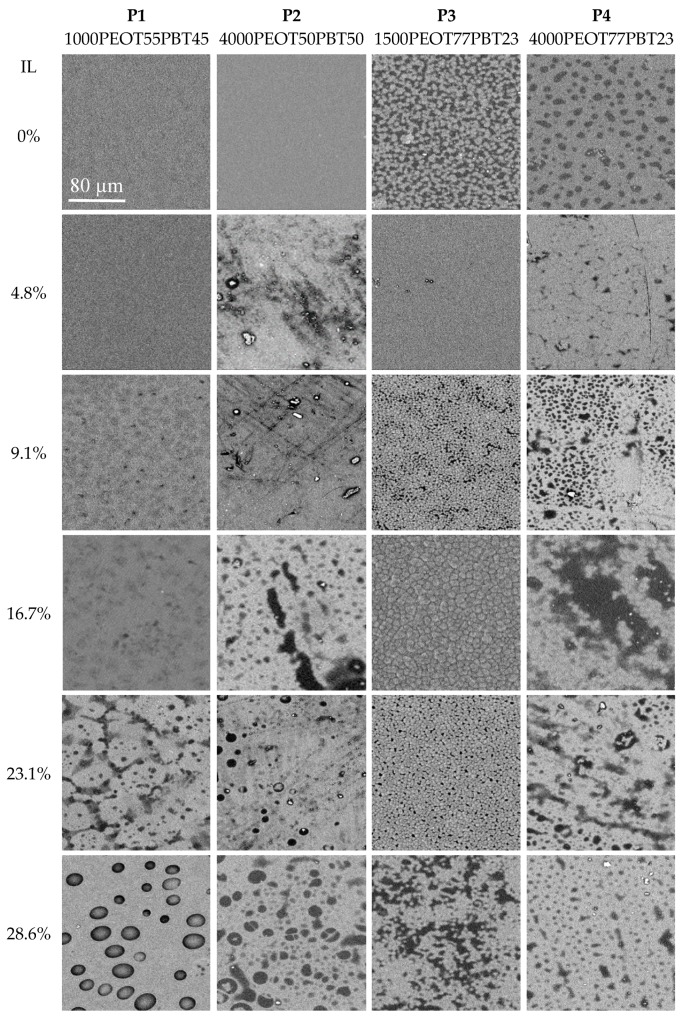
SEM images of the neat polymers (top line) and the polymers with increasing ionic liquid content with backscattering detection, highlighting the ordered crystal phases in the film. All images at magnification 1000×; the scale bar is the same for all samples.

Defect-free membranes were obtained as flexible mechanically stable dense films, having a slightly hazy bulk and a shiny or matt surface (Table 1). Only **P3** with 4.8% IL was fully transparent. No IL exudate was found on the sample surface after the film formation and drying. The haze was caused by the phase separation of the polymer in micrometer-sized crystalline PBT domains and amorphous or semi-crystalline PEOT domains. Anisotropy in the sample was captured by the backscattering detector of the SEM as differences in back-scattered light intensity, where the presence of crystalline structures leads to a higher brightness (Figure 2). Some films had a matt surface due to the formation of micrometer-sized crystals just below the surface, appearing in the SEM analysis as features with a more intense back-scattering, and showing increased surface roughness in the topographic analysis mode (Supplementary Information, Figure S1). Comparing the membranes on the basis of their composition, the neat polymers **P1** and **P2** have a finer microstructure than polymers **P3** and **P4**, which have a much larger PEOT phase than the PBT phase. With increasing ionic liquid content, all samples show increased anisotropy, but this effect is stronger for **P2** and **P4** with their longer PEOT block lengths. Similar observations were made by Yave et al. [16] for the PEO-PBT/PEG-DBE membranes where the PEG-DBE separated phase was seen as brighter areas of islands or hills. Addition of the ionic liquid affects the compatibility of the two block-copolymer phases and, thus, the domain structure development, which is a complex process depending on the relative concentration of all components, on the lengths of the individual blocks, on the solvents used, on the film formation conditions, and other factors [32].

### 3.2. Thermal Properties

**Figure 3:**
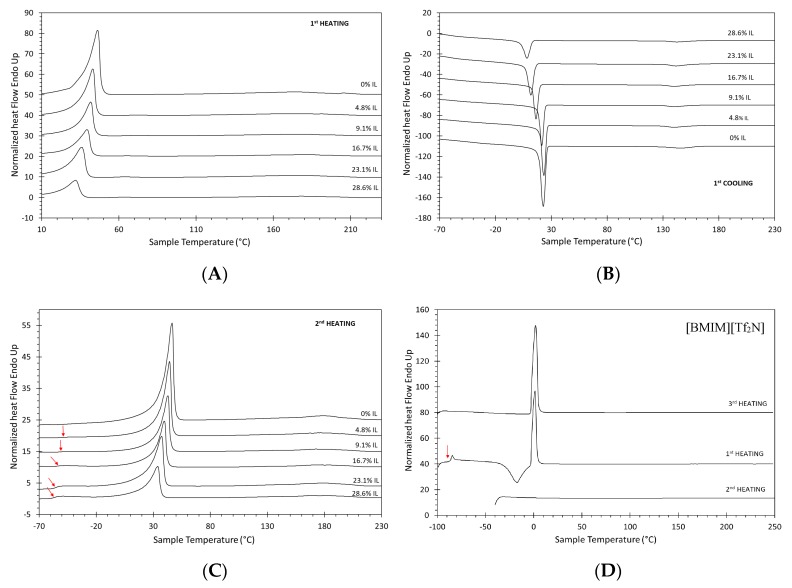
Example of DSC curves of sample **P4** as a function of the ionic liquid (IL) content (in wt.%) at a heating and cooling rate of 15 °C min^−1^. (**A**) First heating run, (**B**) cooling run, (**C**) second heating run. (**D**) DSC curves of the pure ionic liquid [BMIM][Tf_2_N] obtained after different cooling procedures. The curves are shifted vertically for clarity and the Tg is indicated with a red arrow. Program of the analysis of the pure ionic liquid: First heating after normal cooling to −100 °C, second heating after cooling to −40 °C and annealing for 20 min; and third heating after normal cooling to −100°C, heating to −10 °C, annealing for 10 min, followed by a second cooling to −100 °C (see the full temperature program of the IL in Table S1).

All grades of PolyActive^TM^ exhibit microphase separation in a PEOT-rich phase and a PBT-rich phase. A typical example of the DSC curves is shown in Figure 3 for sample **P4** and its blends with ionic liquid at different concentrations. The strong endothermal peak in the range of 30–40 °C and the much smaller peak around 180 °C correspond to the PEOT and the PBT phase, respectively. The endothermal nature in the peaks in the heating run and the exothermal nature in the cooling run, with significant undercooling, confirm that both phases are semi-crystalline. All other grades of PolyActive^TM^ also showed two peaks, corresponding to the PEOT-rich and PBT-rich phase, respectively. The melting enthalpy of PEO and PBT were reported to be 196.8 [33] and 144.5 J/g [34]. The intensity of the peaks was strongly dependent on the molecular weight of the PEOT block, and on the copolymer composition. As the PEOT and the PBT blocks are poorly compatible, the microphase separation of the two phases always occurs, but this phenomenon is strongest for the samples with the highest block length.

The DSC curve of pure sample **P4**, containing a high PEOT weight fraction, shows a strong melting peak around room temperature (Figure 3). Instead, the melting peak of the hard PBT segment in **P4** is very small, and as it is also very broad, it is barely detectable. The butylene terephthalate units are unable to crystalize efficiently, as they are present in low concentrations [34]. Both phases appear as an exothermal peak in the cooling runs with approximately 20 °C undercooling of the PEOT phase and 30 °C undercooling of the PBT phase, before crystallization takes place. In the second heating run, a weak glass transition of the polymer appears at approximately −50 °C. The glass transition becomes more evident at high IL content because the IL reduces crystallinity and increases the amount of amorphous phase. The glass transition temperature of the blend decreases as a function of the IL content, suggesting plasticization of the polymer chains in the presence of the ionic liquid. With a dedicated temperature program, it was possible to suppress the crystallization of the ionic liquid, and it was found that the IL also exhibits a glass transition. Three successive DSC runs were performed on the pure IL (Figure 3D). The first heating curve, after normal cooling at 15 °C min^−1^ to −100 °C, shows a *T*_g_ at around −86 °C, crystallization during heating (around −20 °C), and subsequent melting (around 1 °C). The second heating run, after cooling from 250 °C to −40 °C and subsequent conditioning of the sample at −40 °C for 20 min, shows no phase transitions. Thus, a lower temperature is needed for crystallization. Indeed, conditioning of the sample at −10 °C for 10 min, after it has previously been cooled to −100°C, yields a clear, sharp melting peak in the third heating curve, proving that for nucleation to start, the IL needs to reach a sufficiently low temperature for nucleation to occur and then needs to be kept for a longer time just below the melting temperature. The observed supercooling of the pure IL during the normal heat–cool cycles is generally an indication of its high purity [35], but the complete suppression of crystallization in the polymer is an indication of good molecular-level dispersion.

The simplest model to predict the glass transition temperature of polymer blends or polymers with additives, *T_g_*_, *blend*_, based on the individual components is the well-known Fox equation:

(1) $$\frac{1}{T_{g,blend}}=\frac{w_{pol}}{T_{g,pol}}+\frac{w_{IL}}{T_{g,IL}},$$

where *w_pol_* and *w_IL_* are the weight fractions and *T_g_*_,*pol*_ and *T_g_*_,*IL*_ are the glass transition temperatures of the pure polymer and pure ionic liquid, respectively. More complex models may be needed for block copolymers with different solubilities of the IL in the two phases, but as the *T_g_* of the ionic liquid is lower than that of the neat polymer, the Fox equation correctly describes the qualitative trend of decreasing *T_g_*_,*blend*_ with increasing IL concentration. The ionic liquid itself has a melting point of ca. 1 °C, but none of the blends shows signs of crystallization of the IL. Indeed, even in the pure IL, the crystallization can easily be suppressed, and crystallization is expected to be even more difficult in the case of relatively low concentrations of IL in the blend.

**Figure 4:**
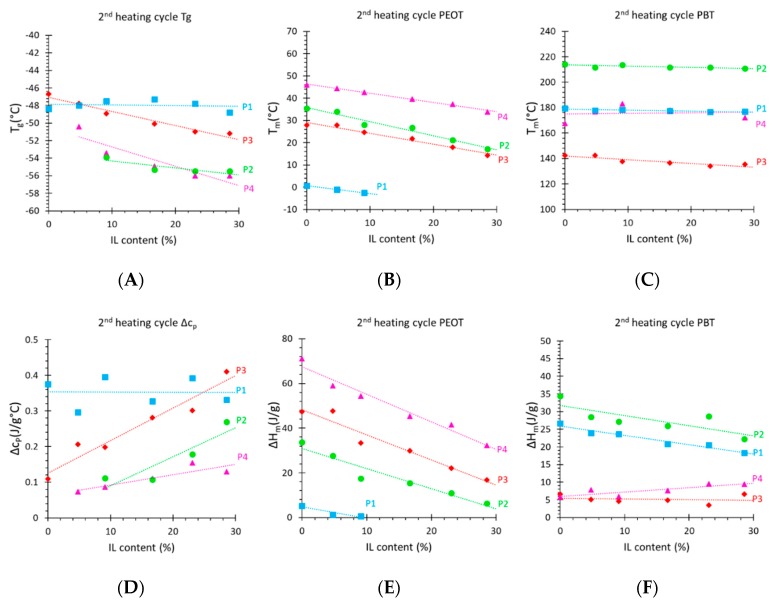
(**A**) Glass transition temperature, (**B**) poly(ethylene oxide terephthalate) (PEOT) phase melting temperature, and (**C**) polybutylene terephthalate (PBT) phase melting temperature. (**D**) Change in specific heat, and (**E**) melting enthalpy of the PEOT phase and (**F**) of the PBT phase.

In the samples with a higher amount of the hard PBT phase, the PBT melting peak becomes more pronounced and sharper (see Figure S4, sample **P2** with 50% PBT). The PBT peak shows double maxima, which is ascribed to the existence of crystals altering in regularity and size [34]. At the same time, the PEOT peak also remains strong in **P2** because the relatively long PEOT block (*M*_w_ = 4000 g mol^−1^) causes efficient microphase separation and allows a higher degree of crystallinity. The *T*_g_ of the PBT phase was not detected in any of the samples, although available literature data report it in the range between 50 and 60 °C [34,36]. The *T_g_* of the soft PEOT phase was visible in samples with lower *M*_w_ of the PEOT phase, due to the less efficient crystallization of the shorter PEOT segments. Further examples of the DSC curves of the blends are shown in Figures S3–S5. An overview of all quantitative data is plotted in Figure 4. There is a significant decrease in the peak area and a slight shift in the PEOT peak position with increasing IL content, but no significant changes in the PBT peak positions. This suggests that IL mixes only with the PEOT phase, but is incompatible with the PBT phase and does not affect it [37]. The numerical values of the thermal analysis are given in Tables S2–S5. The melting enthalpy (∆*H*_m_) of the PBT phase for **P1**, **P2**, and **P3** seems to decrease with increasing IL content, but is constant when normalized for the amount of polymer (Figure S2C). This supports the hypothesis of the complete non-compatibility of the IL and PBT and is visualized by constant peaks of the PBT phase in the DSC curves. The only exception is sample **P4**, where ∆*H_m_* of PBT is increasing with the IL concentration. The explanation of this counterintuitive trend is that the relatively small PBT phase is partially immobilized by the highly rigid crystalline PEOT phase (see mechanical properties below), thus partially suppressing its freedom to crystallize (Figure 4B). However, an increasing amount of IL plasticizes the PEOT phase (Figure 4B) and gives more freedom for the PBT phase to crystalize. ∆*H*_m_ of the PEOT phase (Figure 4E) decreases for all samples, and completely disappears for **P1**, meaning that the PEOT phase becomes fully amorphous.

Qiu et al. [38] reported on the thermal properties of Pebax^®^1657/[BMIM][Tf_2_N] blend membranes with analogous behavior. Herein, the PEO melting peak became smaller and shifted upon addition of the IL, and with a maximum load of 40 wt.% of IL, the PEO peak was no longer detectable by DSC. Additionally, the IL showed good compatibility with the hard PA phase as well, decreasing its crystallinity and enthalpy of fusion. In a study on Pebax^®^1657 and Pebax^®^2533 copolymers doped with [BMIM][CF_3_SO_3_] [29], the IL shows good compatibility with both phases and acts as a plasticizer from the mechanical point of view. However, in Pebax^®^2533, the (normalized) melting enthalpy of both the polyether crystal phase and the polyamide crystal phase remained unchanged even up to 80 wt.% of IL. Figure 4A shows an increasingly evident *T*_g_ of the PEOT phase with the addition of IL. The IL mixes with the PEOT phase and decreases its crystallinity, but it evidently does not significantly affect the *T*_g_ of the polymer, which could suggest strong interactions between IL and the polymer, and explain the limited validity of the Fox equation (Equation (1)). The melting temperature (*T*_m_) of PBT is the highest for sample **P2** with the highest PBT content, embedded in PEOT with long-chain segments, and smallest for sample **P3** with the lowest content of PBT and also low *M*_w_ of PEOT. The *T*_m_ of PEOT follows the same trend. A high fraction of the PEOT phase in sample **P4** and its high *M*_w_ (4000 g mol^−1^) results in the highest *T*_m_, opposite to sample **P1** with a small fraction and low *M*_w_ of the PEOT phase. Addition of IL results in a decrease in *T*_m_ of the PEOT phase for all samples apparently because of the higher interaction of the IL with the PEOT phase. Thus, the PBT melting temperature increases both with increasing PBT content and with increasing PEOT block length, but it is remarkably independent of the IL concentration. This indicates that a higher PEOT block length favors more efficient microphase separation into PEOT and PBT domains and that the IL does not affect the PBT phase.

### 3.3. Mechanical Properties

**Figure 5:**
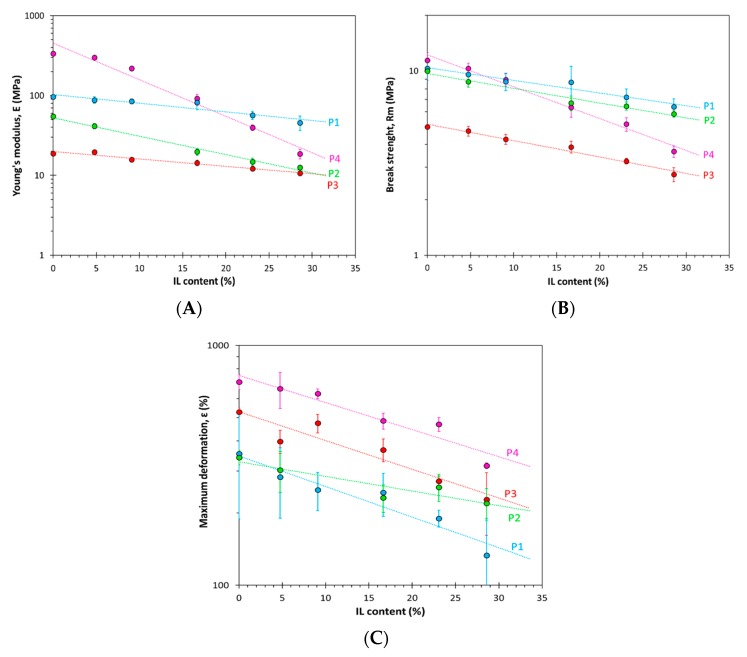
(**A**) Young’s modulus, (**B**) tensile strength, and (**C**) maximum elongation of the PolyActive^TM^/IL membranes as a function of the IL content. Lines are shown as a guide to the eye. Points and error bars indicate the average and standard deviation of 4–5 specimens of each membrane sample.

All the prepared membranes have typical rubbery behavior, characterized by low modulus and high elongation (Figure 5). The neat **P4** polymer has the highest Young’s modulus, which drastically decreases with increasing IL concentration, showing that this IL acts as a strong plasticizer for this grade of PolyActive^TM^ having the lowest amount of hard PBT blocks and the highest PEOT block length. The drastic decrease in Young’s modulus and break strength with increasing IL content in **P4** is mostly a result of the decrease in the crystallinity of the PEOT phase, as was confirmed by DSC analysis (Figure 4E). In addition, the **P2** series, which has a similar *M*_w_ of PEOT but a higher fraction of PBT, shows a significant decline in the elastic modulus as a function of the IL content. Therefore, it can be concluded that a higher M_w_ of soft phase will enable more efficient crystallization, which will give better mechanical properties to the membrane, in accordance with previously reported work [36]. At the same time, these polymers will be affected most by the presence of the IL. **P1** and **P3** are less affected by the presence of the IL due to the low *M*_w_ of their PEOT domains. Sample **P1** has a higher modulus and break strength than sample **P2**, with similar content of the PBT hard segments, but with higher *M*_w_ of the PEOT blocks and higher ∆*H*_m_ of both phases. Apparently, the stronger microphase separation in **P2** leads to relatively poor mechanical properties. For entropic reasons, low-molecular-weight compounds mix better with each other and, similarly, polymers with shorter block lengths tend to be more compatible and the phase separation between the soft and hard segment will decrease with the lower *M*_w_ of the PEOT phase [36,39], where inter-chain interactions become more important than intra-chain interactions. As inter-chain interactions are relevant for network formation, this will result in increased mechanical strength and elasticity of the sample [29]. Maximum deformation is higher in the more flexible **P4** grade (high *M*_w_ of PEOT and high fraction), and then follows the trend **P3** (low *M*_w_ of PEOT but high fraction) > **P2** (high *M*_w_ of PEOT but low fraction) > **P1** (low *M*_w_ of PEOT and low fraction) over the entire composition range, and it decreases with IL content for all four grades of PolyActive^TM^.

## 4. Conclusions

The correlation between the amount of the ionic liquid [BMIM][Tf_2_N] and the morphological, thermal, and mechanical properties of four different grades of PolyActive™ poly(ether-ester) multiblock copolymer was studied. The IL was found to dissolve mainly into the PEOT phase, reducing the crystallinity of the PEOT segments and inducing the appearance of an amorphous phase with the corresponding glass transition. Without any exception, the ionic liquid caused a gradual decrease in the mechanical properties in terms of Young’s modulus, the tensile strength, and maximum elongation in all samples. It also causes increased anisotropy in the films, up to the formation of droplet-like domains in the PBT-rich samples. The negative effect on Young’s modulus was strongest for the samples with long PEOT blocks due to the relatively strong reduction in the crystallinity of the PEOT phase in these samples. The mechanical properties are consistent with the results of the thermal analysis, giving a complete picture of how the properties of the PolyActive™-based blends can be tailored by the characteristics of the PolyActive™ grade itself, and the amount of ionic liquid dispersed therein. The PBT melting temperature increases both with increasing PBT content and with increasing PEOT block length in the copolymer, but it is remarkably independent of the IL concentration. This indicates that a higher PEOT block length favors a more efficient microphase separation into PEOT- and PBT-rich domains and that the IL does not affect the PBT phase. The IL therefore affects mostly the PEOT phase. All films appear dry on the surface and are mechanically stable, suggesting potential use as packaging films, gas separation membranes, or as ion-conducting layers in batteries. Further studies on the potential application of the films as gas separation membranes will be the subject of our future work. We expect that the gained knowledge on material behavior and properties will lead to the fabrication of efficient PolyActive™/ [BMIM][Tf_2_N] blend membranes for effective CO_2_ separation from hydrogen, methane, or other gas mixtures. This work is in progress and will be the subject of a future publication.

## Figures and Tables

**Table 1:** Membrane compositions and macroscopic properties.

	Polymer ^a^			[BMIM][Tf_2_N]	Membrane	
Sample Code	M_PEO_ (g mol^−1^)	W_PEOT_ (wt.%)	W_PBT_ (wt.%)	Concentration wt.% ^d^	Thickness (µm) ^e^	Appearance
1000PEOT55PBT45	1000	55	45	0	103	Shiny
**(P1)** ^b^				4.8	182	Shiny
				9.1	121	Shiny
				16.7	115	Shiny
				23.1	127	Shiny
				28.6	192	Matt
4000PEOT50PBT50	4000	50	50	0	81.5	Shiny
**(P2)** ^c^				4.8	95.3	Shiny
				9.1	129	Shiny
				16.7	105	Shiny
				23.1	95	Shiny
				28.6	109	Shiny
1500PEOT77PBT23	1500	77	23	0	121	Matt
**(P3)** ^b^				4.8	107	Transparent
				9.1	100	Matt
				16.7	142	Matt
				23.1	134	Matt
				28.6	164	Matt
4000PEOT77PBT23	4000	77	23	0	108	Matt
**(P4)** ^b^				4.8	106	Matt
				9.1	130	Matt
				16.7	139	Matt
				23.1	160	Matt
				28.6	161	Matt

^a^ M_PEO_ = molar mass of the PEO block, W_PEOT_ = weight fraction of PEOT blocks, W_PBT_ = weight fraction of PBT blocks. ^b^ Prepared from a 5 wt.% solution in chloroform at 25 °C, unless specified otherwise. ^c^ Prepared from a 3 wt.% solution under reflux (ca. 60 °C). ^d^ IL concentration based on the final sample weight, assuming complete evaporation of the solvent. ^e^ Sample thickness determined with a Mitutoyo digital micrometer as an average of 10 spots.

## Supplementary Materials

The following are available online at https://www.mdpi.com/2073-4360/12/4/890/s1, Comprehensive file containing **Figure S1:** Example of SEM images of sample **P2** and sample **P4** with 20 wt.% IL, **Table S1:** Temperature cycle of the pure [BMIM][Tf_2_N] sample for DSC analysis, **Table S2:** Thermal properties of the PolyActive™ **P1**/[BMIM][Tf_2_N] blend membranes, **Table S3:** Thermal properties of the PolyActive™ **P2**/[BMIM][Tf_2_N] blend membranes, **Table S4:** Thermal properties of the PolyActive™ **P3**/[BMIM][Tf_2_N] blend membranes, **Table S5:** Thermal properties of the PolyActive™ **P4**/[BMIM][Tf_2_N] blend membranes, **Figure S2:** A) Normalized change in specific heat, B) normalized melting enthalpy of the PEOT phase, and C) normalized melting enthalpy of the PBT phase, **Figure S3:** DSC curves of sample **P1** as a function of the IL content, **Figure S4:** DSC curves of sample **P2** as a function of the IL content, **Figure S5:** DSC curves of sample **P3** as a function of the IL content, **Figure S6:** Example of the tensile curves of sample **P4**.

## References

[1] Vermonden T., Censi R., Hennink W.E. (2012). Hydrogels for protein delivery. Chem. Rev..

[2] Hahn J. (2009). Biopharmaceuticals-challenges and opportunities facing the drug-delivery industry. Touch Brief..

[3] Waris E., Ashammakhi N., Lehtimäki M., Tulamo R.-M., Törmälä P., Kellomäki M., Konttinen Y.T. (2008). Long-term bone tissue reaction to polyethylene oxide/polybutylene terephthalate copolymer (Polyactive^®^) in metacarpophalangeal joint reconstruction. Biomaterials.

[4] Claase M.B., Grijpma D.W., Mendes S.C., de Bruijn J.D., Feijen J. (2003). Porous PEOT/PBT scaffolds for bone tissue engineering: Preparation, characterization, and in vitro bone marrow cell culturing. J. Biomed. Mater. Res. Part A Off. J. Soc. Biomater..

[5] Schuldt K., Pohlmann J., Shishatskiy S., Brinkmann T. (2018). Applicability of PolyActive™ Thin Film Composite Membranes for CO_2_ Separation from C_2_H_4_ Containing Multi-Component Gas Mixtures at Pressures up to 30 Bar. Membranes.

[6] Karunakaran M., Shevate R., Kumar M., Peinemann K.-V. (2015). CO_2_-selective PEO–PBT (PolyActive™)/graphene oxide composite membranes. Chem. Commun..

[7] Lin H., Freeman B.D. (2005). Materials selection guidelines for membranes that remove CO_2_ from gas mixtures. J. Mol. Struct..

[8] Lin H., Freeman B.D. (2004). Gas solubility, diffusivity and permeability in poly(ethylene oxide). J. Membr. Sci..

[9] Liu S.L., Shao L., Chua M.L., Lau C.H., Wang H., Quan S. (2013). Recent progress in the design of advanced PEO-containing membranes for CO_2_ removal. Prog. Polym. Sci..

[10] Bondar V., Freeman B.D., Pinnau I. (2000). Gas transport properties of poly(ether-b-amide) segmented block copolymers. J. Polym. Sci. Part B Polym. Phys..

[11] Car A., Stropnik C., Yave W., Peinemann K.V. (2008). Tailor-made polymeric membranes based on segmented block copolymers for CO_2_ separation. Adv. Funct. Mater..

[12] Fakirov S., Apostolov A., Boeseke P., Zachmann H. (1990). Structure of segmented poly(ether ester) s as revealed by synchrotron radiation. J. Macromol. Sci. Part B Phys..

[13] Barbi V., Funari S.S., Gehrke R., Scharnagl N., Stribeck N. (2003). SAXS and the Gas Transport in Polyether-b lock-polyamide Copolymer Membranes. Macromolecules.

[14] Metz S., Mulder M., Wessling M. (2004). Gas-permeation properties of poly(ethylene oxide) poly(butylene terephthalate) block copolymers. Macromolecules.

[15] Car A., Stropnik C., Yave W., Peinemann K.-V. (2008). PEG modified poly(amide-b-ethylene oxide) membranes for CO_2_ separation. J. Membr. Sci..

[16] Yave W., Car A., Funari S.S., Nunes S.P., Peinemann K.-V. (2009). CO_2_-philic polymer membrane with extremely high separation performance. Macromolecules.

[17] Rahman M.M., Filiz V., Shishatskiy S., Abetz C., Georgopanos P., Khan M.M., Neumann S., Abetz V. (2015). Influence of poly(ethylene glycol) segment length on CO_2_ permeation and stability of polyactive membranes and their nanocomposites with PEG POSS. ACS Appl. Mater. Interfaces.

[18] Rahman M.M., Filiz V., Shishatskiy S., Abetz C., Neumann S., Bolmer S., Khan M.M., Abetz V. (2013). PEBAX^®^ with PEG functionalized POSS as nanocomposite membranes for CO_2_ separation. J. Membr. Sci..

[19] Reijerkerk S.R., Knoef M.H., Nijmeijer K., Wessling M. (2010). Poly(ethylene glycol) and poly(dimethyl siloxane): Combining their advantages into efficient CO_2_ gas separation membranes. J. Membr. Sci..

[20] Lillepärg J., Georgopanos P., Shishatskiy S. (2014). Stability of blended polymeric materials for CO_2_ separation. J. Membr. Sci..

[21] Murali R.S., Ismail A., Rahman M., Sridhar S. (2014). Mixed matrix membranes of Pebax-1657 loaded with 4A zeolite for gaseous separations. Sep. Purif. Technol..

[22] Zheng W., Ding R., Yang K., Dai Y., Yan X., He G. (2019). ZIF-8 nanoparticles with tunable size for enhanced CO_2_ capture of Pebax based MMMs. Sep. Purif. Technol..

[23] Sabetghadam A., Liu X., Gottmer S., Chu L., Gascon J., Kapteijn F. (2019). Thin mixed matrix and dual layer membranes containing metal-organic framework nanosheets and Polyactive™ for CO_2_ capture. J. Membr. Sci..

[24] Liu M., Xie K., Nothling M.D., Gurr P.A., Tan S.S.L., Fu Q., Webley P.A., Qiao G.G. (2018). Ultrathin Metal–Organic Framework Nanosheets as a Gutter Layer for Flexible Composite Gas Separation Membranes. ACS Nano.

[25] Dai Z., Bai L., Hval K.N., Zhang X., Zhang S., Deng L. (2016). Pebax^®^/TSIL blend thin film composite membranes for CO_2_ separation. Sci. China Chem..

[26] Noble R.D., Gin D.L. (2011). Perspective on ionic liquids and ionic liquid membranes. J. Membr. Sci..

[27] Kohoutová M., Sikora A., Hovorka Š., Randová A., Schauer J., Tišma M., Setničková K., Petričkovič R., Guernik S., Greenspoon N. (2009). Influence of ionic liquid content on properties of dense polymer membranes. Eur. Polym. J..

[28] Baltus R.E., Counce R.M., Culbertson B.H., Luo H., DePaoli D.W., Dai S., Duckworth D.C. (2005). Examination of the potential of ionic liquids for gas separations. Sep. Sci. Technol..

[29] Bernardo P., Jansen J.C., Bazzarelli F., Tasselli F., Fuoco A., Friess K., Izák P., Jarmarová V., Kačírková M., Clarizia G. (2012). Gas transport properties of Pebax^®^/room temperature ionic liquid gel membranes. Sep. Purif. Technol..

[30] Estahbanati E.G., Omidkhah M., Amooghin A.E. (2017). Preparation and characterization of novel Ionic liquid/Pebax membranes for efficient CO_2_/light gases separation. J. Ind. Eng. Chem..

[31] Jansen J.C., Friess K., Clarizia G., Schauer J., Izak P. (2011). High ionic liquid content polymeric gel membranes: Preparation and performance. Macromolecules.

[32] Bates F.S., Fredrickson G.H. (1990). Block copolymer thermodynamics: Theory and experiment. Ann. Rev. Phys. Chem..

[33] Silva V., Silva G., Caliman V., Rieumont J., de Miranda-Pinto C., Archanjo B., Neves B. (2007). Morphology, crystalline structure and thermal properties of PEO/MEEP blends. Eur. Polym. J..

[34] Fakirov S., Gogeva T. (1990). Poly(ether/ester) s based on poly(butylene terephthalate) and poly(ethylene glycol), 1. Poly(ether/ester) s with various polyether: Polyester ratios. Die Makromol. Chem. Macromol. Chem. Phys..

[35] Esperança J.M., Tariq M., Pereiro A.B., Araújo J.M., Seddon K.R., Rebelo L.P.N. (2019). Anomalous and Not-So-Common Behaviour in Common Ionic Liquids and Ionic Liquid-containing Systems. Front. Chem..

[36] Deschamps A.A., Grijpma D.W., Feijen J. (2001). Poly(ethylene oxide)/poly(butylene terephthalate) segmented block copolymers: The effect of copolymer composition on physical properties and degradation behavior. Polymer.

[37] Miranda D.F., Russell T.P., Watkins J.J. (2010). Ordering in mixtures of a triblock copolymer with a room temperature ionic liquid. Macromolecules.

[38] Qiu Y., Ren J., Zhao D., Li H., Deng M. (2016). Poly(amide-6-b-ethylene oxide)/[Bmim][Tf2N] blend membranes for carbon dioxide separation. J. Energy Chem..

[39] Sonpatki M., Ravindranath K., Ponrathnam S. (1994). Random thermotropic elastomers. I. Effects of substitution and hard/soft segment lengths on properties. J. Polym. Sci. Part A Polym. Chem..

