# Supplementary material for: Tailoring the Thermal and Mechanical Properties of PolyActiveTM Poly(Ether-Ester) Multiblock Copolymers Via Blending with CO2-Phylic Ionic Liquid
Source: Polymers (Basel). 2020 Apr 12;12(4):890. doi: 10.3390/polym12040890 (PMC7240668; doi:10.3390/polym12040890)
Supplement: Supplementary file 1 [file polymers-12-00890-s001.pdf]

Supporting information

Selected experimental details and results for the manuscript “Tailoring the properties of PolyActive™ poly(ether-ester) multiblock copolymers via blending with ionic liquid.”

Tailoring the properties of PolyActive™ poly(ether-ester) multiblock copolymers via blending with ionic liquids

Martina Klepić [1], Alessio Fuoco [2], Marcello Monteleone [2], Elisa Esposito [2,*], Karel Friess [1,3], Zuzana Petrusová [3], Pavel Izák [1,3] and Johannes Carolus Jansen [2,*]

[1] Department of Physical Chemistry, University of Chemistry and Technology Prague, Technická 5, 166 28 Prague 6, Czech Republic; mklepic0@gmail.com (M.K.); karel.friess@vscht.cz (K.F.); izak@icpf.cas.cz (P.I.)

[2] Institute on Membrane Technology (CNR-ITM), Via P. Bucci, 17/C, 87036 Rende (CS), Italy; a.fuoco@itm.cnr.it (A.F.); m.monteleone@itm.cnr.it (M.M.)

[3] Czech Academy of Sciences, Institute of Chemical Process Fundamentals, Rozvojová 135, 165 02 Prague 6—Suchdol, Czech Republic; petrusova@icpf.cas.cz (Z.P.)

* Correspondence: e.esposito@itm.cnr.it (E.E.), johannescarolus.jansen@cnr.it (J.C.J.)

Scanning electron microscopy

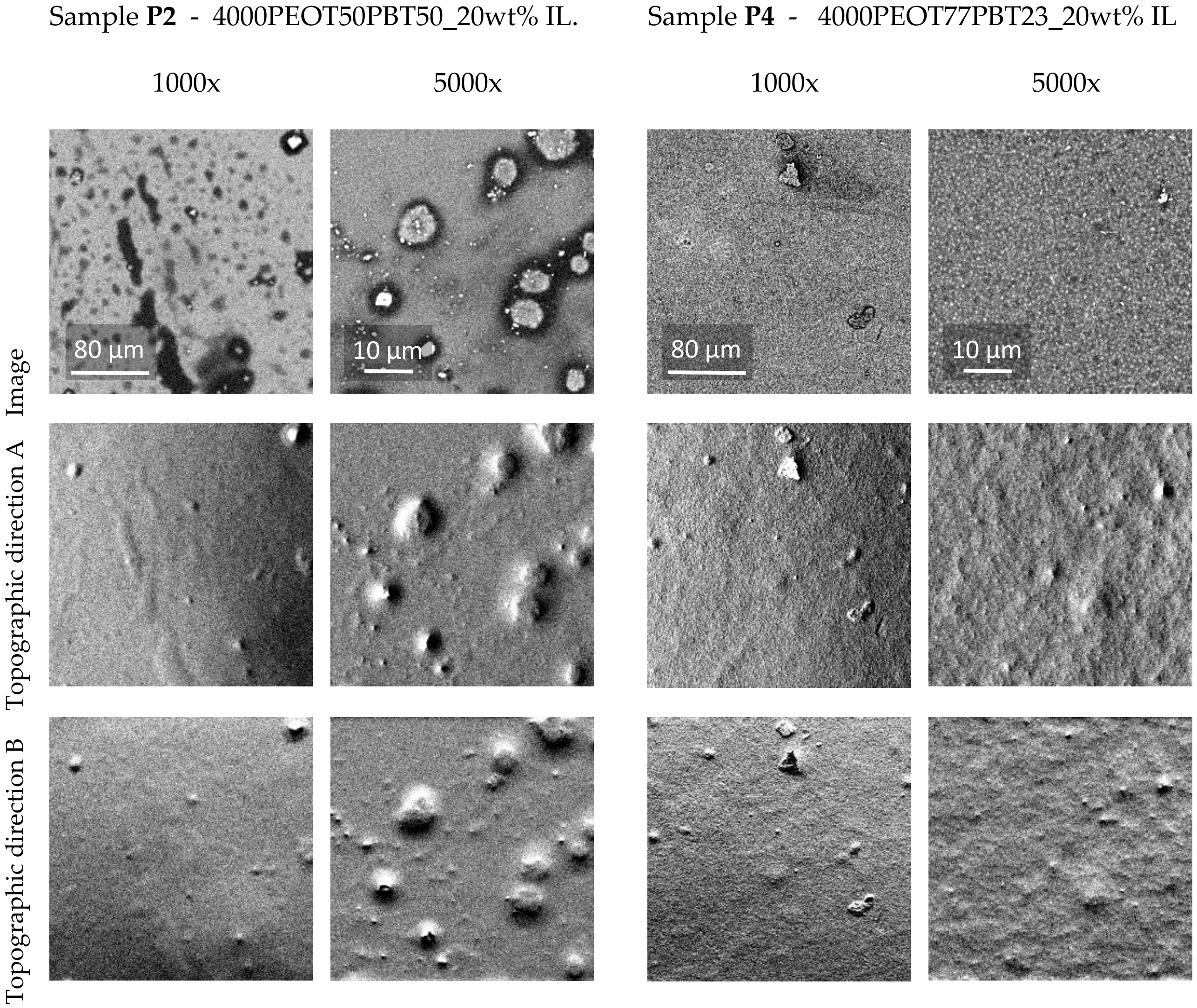

Figure S 1. Example of SEM images of sample **P2** and sample **P4** with 20wt% IL, in two areas with different magnifications. Images recorded in normal imaging mode (left) and in topographic mode, in perpendicular directions (middle and right), highlighting the surface roughness and embedded anisotropies with different backscattering intensity. Irradiation mode: point. The scale bars are identical for all photos at the same magnification.

Differential scanning calorimetry

Table S1. Temperature cycle of the pure [BMIM][Tf_2N] sample for DSC analysis.

Run	Mode	Heating/Cooling			Isothermal		Notes
		Start T (°C)	End T (°C)	Rate (°C min^{-1})	T (°C)	Time (min)	
1	heat	0	250	15			
	isothermal				250	1	
	cool	250	-100	15			
	isothermal				-100	10	
	heat	-100	250	15			Figure 3D, curve 1
	cool	250	-100	Max.			
2	heat	-100	250	15			
	isothermal				250	1	
	cool	250	-40	15			
	isothermal				-40	20	
	heat	-40	250	15			Figure 3D, curve 2
	isothermal				250	1	
	cool	250	-100	15			
	isothermal				-100	1	
	heat	-100	-10	15			
	isothermal				-10	10	
	cool	-10	-100	15			
3	heat	-100	250	15			Figure 3D, curve 3

Table S2. Thermal properties of the PolyActive™ **P1**/[BMIM][Tf_2N] blend membranes

		Glass transition		PEOT melting peak		PBT melting peak	
Run [a)]	IL content (wt%)	T_g (°C)	dC_p (J g^{-1} °C^{-1})	T_m (°C)	dH_m (J g^{-1})	T_m (°C)	dH_m (J g^{-1})
First heating	0			69.3	1.75	176.8	34.97
	4.8			69.8	1.81	177.8	28.13
	9.1			70.5	1.99	177.8	28
	16.7			70.6	1.76	176.3	24.5
	23.1			72.3	1.64	177.3	23.6
	28.6			72.6	1.59	176.1	21
Cooling	0					135.6	-24.0
	4.8					132.3	-21.8
	9.1					137.1	-22.4
	16.7					135.3	-20.2
	23.1					135.9	-18.8
	28.6					135.6	-16.8
Second heating	0	-48.4	0.375	0.68	5.2	179.5	26.6
	4.8	-48.0	0.296	-1.07	1.28	177.7	23.9
	9.1	-47.5	0.395	-2.58	0.612	178.2	23.6
	16.7	-47.3	0.327			177.5	20.8
	23.1	-47.8	0.392			176.7	20.5
	28.6	-48.8	0.331			177.0	18.3

[a)] DSC program: heating 0 - 250°C at 15°C min^{-1}; isothermal 1 min; cooling 250 - -100°C at -15°C min^{-1}; isothermal 1 min; heating 0 - 250°C at 15°C min^{-1}.

Table S3. Thermal properties of the PolyActive™ **P2**/[BMIM][Tf_2N] blend membranes

		Glass transition		PEOT melting peak		PBT melting peak	
		T_g	dC_p	T_m	dH_m	T_m	dH_m
Run [a)]	IL content (wt%)	(°C)	(J g^{-1} °C^{-1})	(°C)	(J g^{-1})	(°C)	(J g^{-1})
First heating	0			35.6	28.9	222.6	43.8
	4.8			33.6	24.2	222.6	38.8
	9.1			28.4	12.3	222.6	36.2
	16.7			27.3	10.2	222.3	33.7
	23.1			23.8	2.19	222.8	36.8
	28.6					222.9	29.2
Cooling	0			11.1	-29.1	178.6	-29.1
	4.8			9.89	-26.2	180.4	-28.1
	9.1			1.44	-17.5	179.4	-24.5
	16.7			-0.591	-15.6	179.6	-24.1
	23.1			-12.3	-8.3	178.9	-24.1
	28.6					177.9	-21.5
Second heating	0			35.3	33.6	214.2	34.4
	4.8			34.0	27.6	211.7	28.5
	9.1	-53.9	0.111	28.0	17.4	213.7	27.2
	16.7	-55.3	0.107	26.8	15.4	211.5	25.9
	23.1	-55.5	0.178	21.2	10.9	211.7	28.7
	28.6	-55.5	0.269	17.2	6.23	210.7	22.2

[a)] DSC program: heating 0 - 250°C at 15°C min^{-1}; isothermal 1 min; cooling 250 - -100°C at -15°C min^{-1}; isothermal 1 min; heating 0 - 250°C at 15°C min^{-1}.

Table S4. Thermal properties of the PolyActive™ **P3**/[BMIM][Tf_2N] blend membranes

		Glass transition		PEOT melting peak		PBT melting peak	
Run [a)]	IL content (wt%)	T_g (°C)	dC_p ($J\ g^{-1}\ °C^{-1}$)	T_m (°C)	dH_m ($J\ g^{-1}$)	T_m (°C)	dH_m ($J\ g^{-1}$)
First heating	0	24.4	33.6	57.5	1.0	134.8	4.7
	4.8	19.3	23.6	55.2	1.7	129.2	7.3
	9.1	23.8	23.3	59.5	1.1	140.6	4.6
	16.7	21	19.5	62.0	1.19	135.3	4.96
	23.1	16.5	8.98	61.5	1.28	140.6	2.1
	28.6	15.4	1.32	67.9	1.39	134.4	5.54
Cooling	0			6.41	-40.3	103.8	-6.99
	4.8			4.19	-37.3	91.9	-10.1
	9.1			2.54	-27.2	86.5	-7.98
	16.7			-1.45	-24.1	86.2	-8.11
	23.1			-6.93	-17.5	86.0	-7.77
	28.6			-14.8	-12	82.5	-6.47
Second heating	0	-46.7	0.109	27.8	47.4	142.3	6.65
	4.8	-47.8	0.206	27.8	47.6	142.5	5.1
	9.1	-48.9	0.198	24.7	33.4	137.7	4.56
	16.7	-50.1	0.281	21.7	29.8	136.5	4.94
	23.1	-51	0.301	18.0	22.0	134.0	3.49
	28.6	-51.2	0.41	14.3	16.8	135.5	6.61

[a)] DSC program: heating 0 - 250°C at 15°C min^{-1}; isothermal 1 min; cooling 250 - -100°C at -15°C min^{-1}; isothermal 1 min; heating 0 - 250°C at 15°C min^{-1}.

Table S5. Thermal properties of the PolyActive™ **P4**/[BMIM][Tf_2N] blend membranes

		Glass transition		PEOT melting peak		PBT melting peak	
		T_g	dC_p	T_m	dH_m	T_m	dH_m
Run [a)]	IL content (wt%)	(°C)	(J g^{-1} °C^{-1})	(°C)	(J g^{-1})	(°C)	(J g^{-1})
First heating	0			46	68.1	168.3	2.56
	4.8			43.2	58.4	179.4	10.2
	9.1			41.7	51.2	178.6	7.58
	16.7			39.4	44.9	181.1	8.29
	23.1			35.9	35.9	177.1	9.81
	28.6			31.9	27.1	178.8	8.37
Cooling	0			21.3	-64.6	151.0	-3.15
	4.8			23.3	-61.3	139.9	-8.19
	9.1			21.5	-54.4	140.1	-5.48
	16.7			16.2	-44.3	139.9	-5.91
	23.1			11.7	-37.6	141.6	-6.93
	28.6			8.17	-28.3	142.6	-6.5
Second heating	0			45.9	71.1	167.7	5.76
	4.8	-50.4	0.0738	44.4	58.9	176.8	7.84
	9.1	-53.4	0.087	42.6	54.3	183.1	5.87
	16.7	-54.9	0.11	39.6	45.3	178.0	7.60
	23.1	-56	0.154	37.3	41.4	176.3	9.51
	28.6	-56	0.129	33.8	32.2	172.2	9.47

[a)] DSC program: heating 0 - 250°C at 15°C min^{-1}; isothermal 1 min; cooling 250 - -100°C at -15°C min^{-1}; isothermal 1 min; heating 0 - 250°C at 15°C min^{-1}.

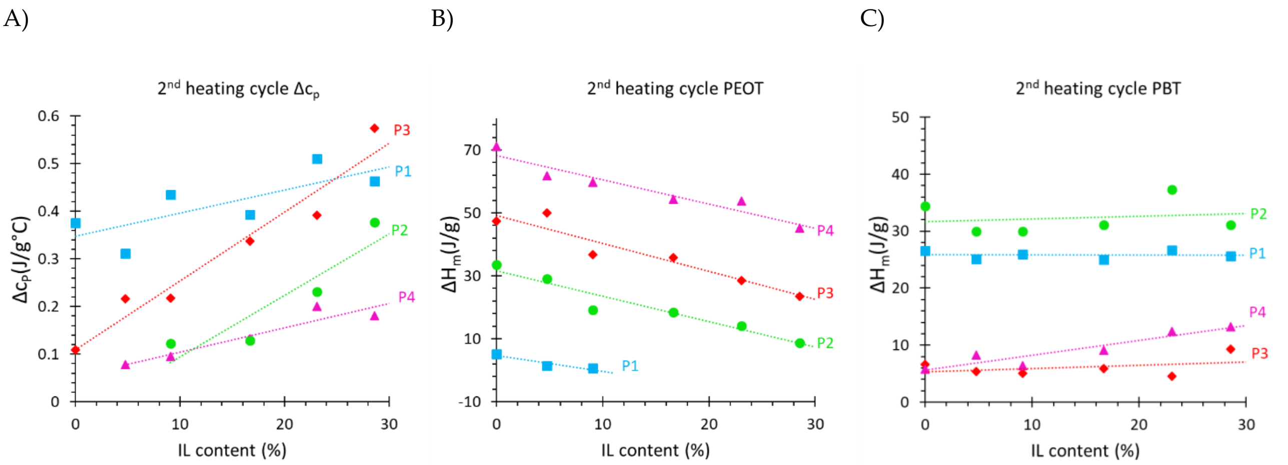

Figure S2. A) Normalized change in specific heat, B) Normalized melting enthalpy of the PEOT phase and C) Normalized melting enthalpy of the PBT phase. Normalization is based on the amount of polymer in the blend.

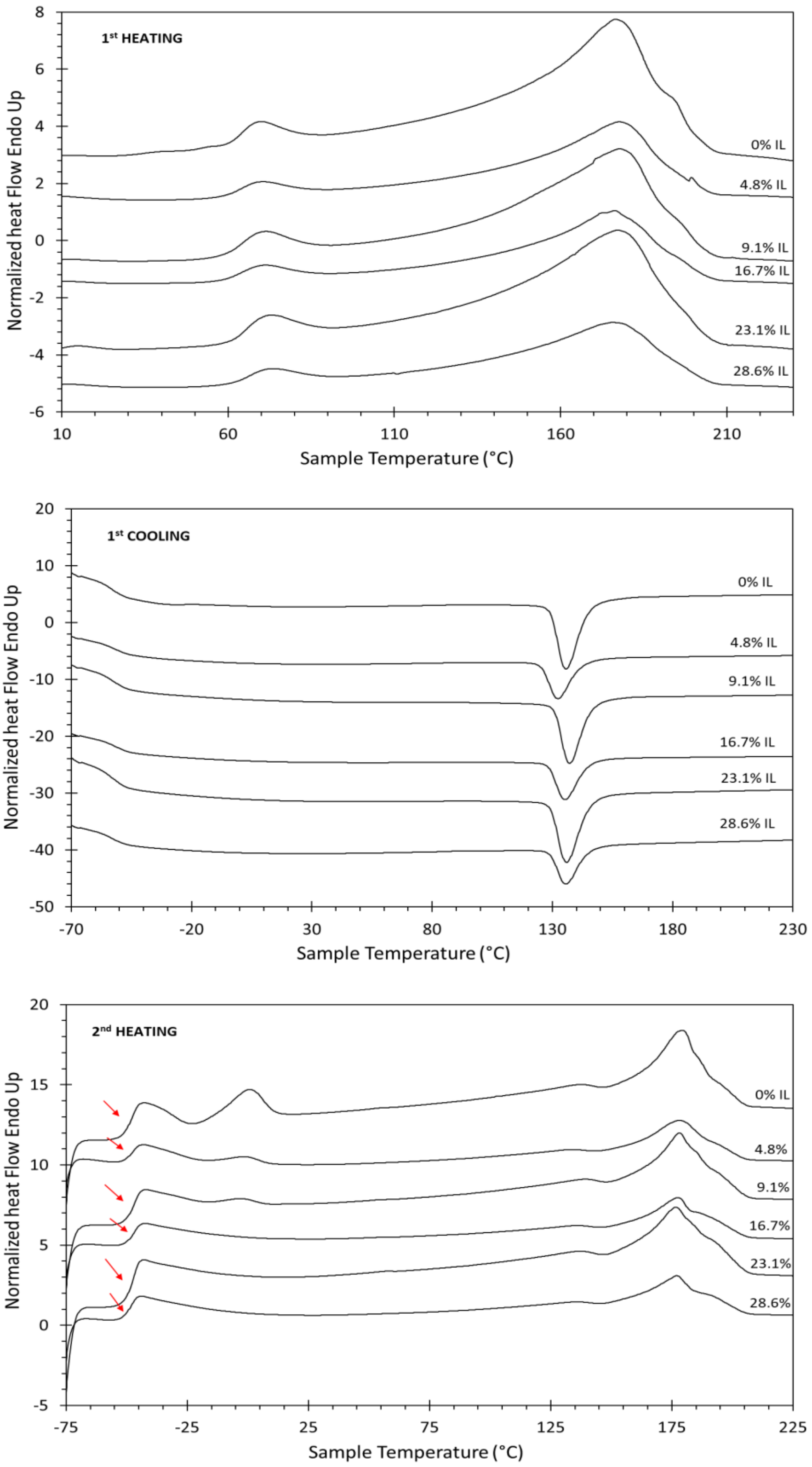

Figure S3. DSC curves of sample **P1** as a function of the IL content (in wt%). The curves are shifted vertically for clarity. The T_g is indicated with red arrows. Heating and cooling rate 15 °C min^{-1}.

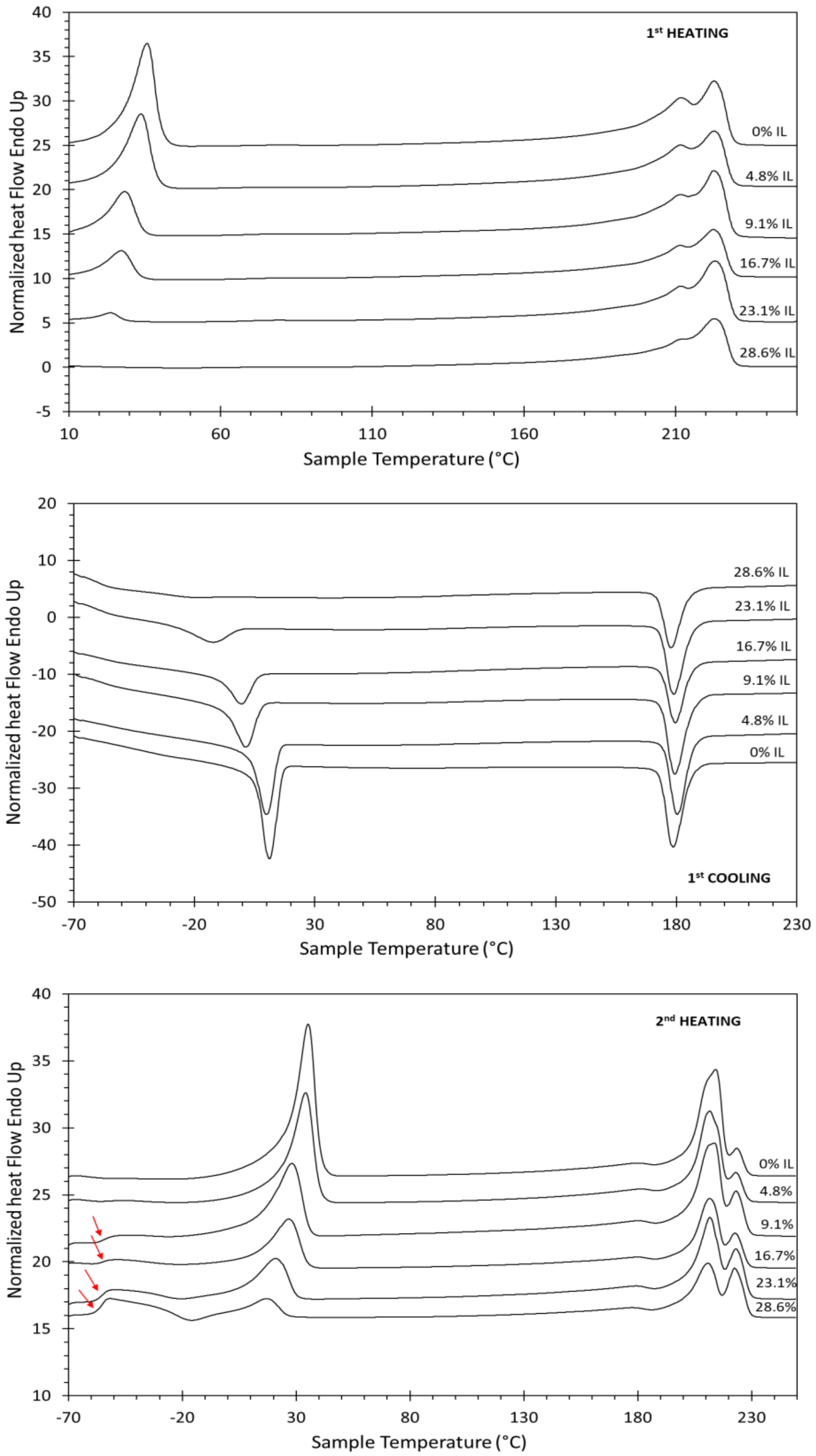

Figure S4. DSC curves of sample **P2** as a function of the IL content (in wt%). The curves are shifted vertically for clarity. The T_g is indicated with red arrows. Heating and cooling rate 15 °C min^{-1}.

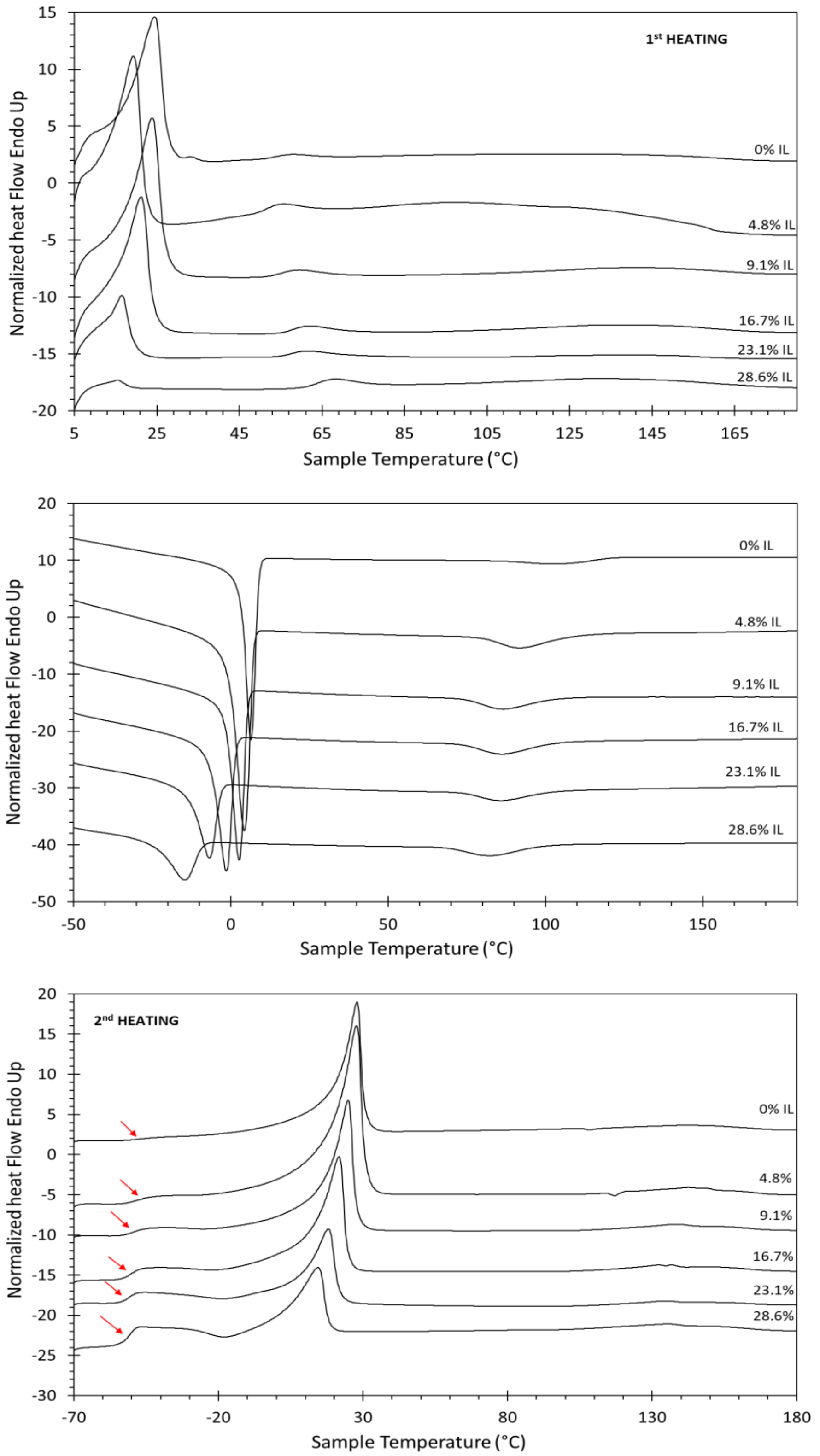

Figure S5. DSC curves of sample **P3** as a function of the IL content (in wt%). The curves are shifted vertically for clarity. The T_g is indicated with red arrows. Heating and cooling rate 15 °C min^{-1}.

Tensile tests

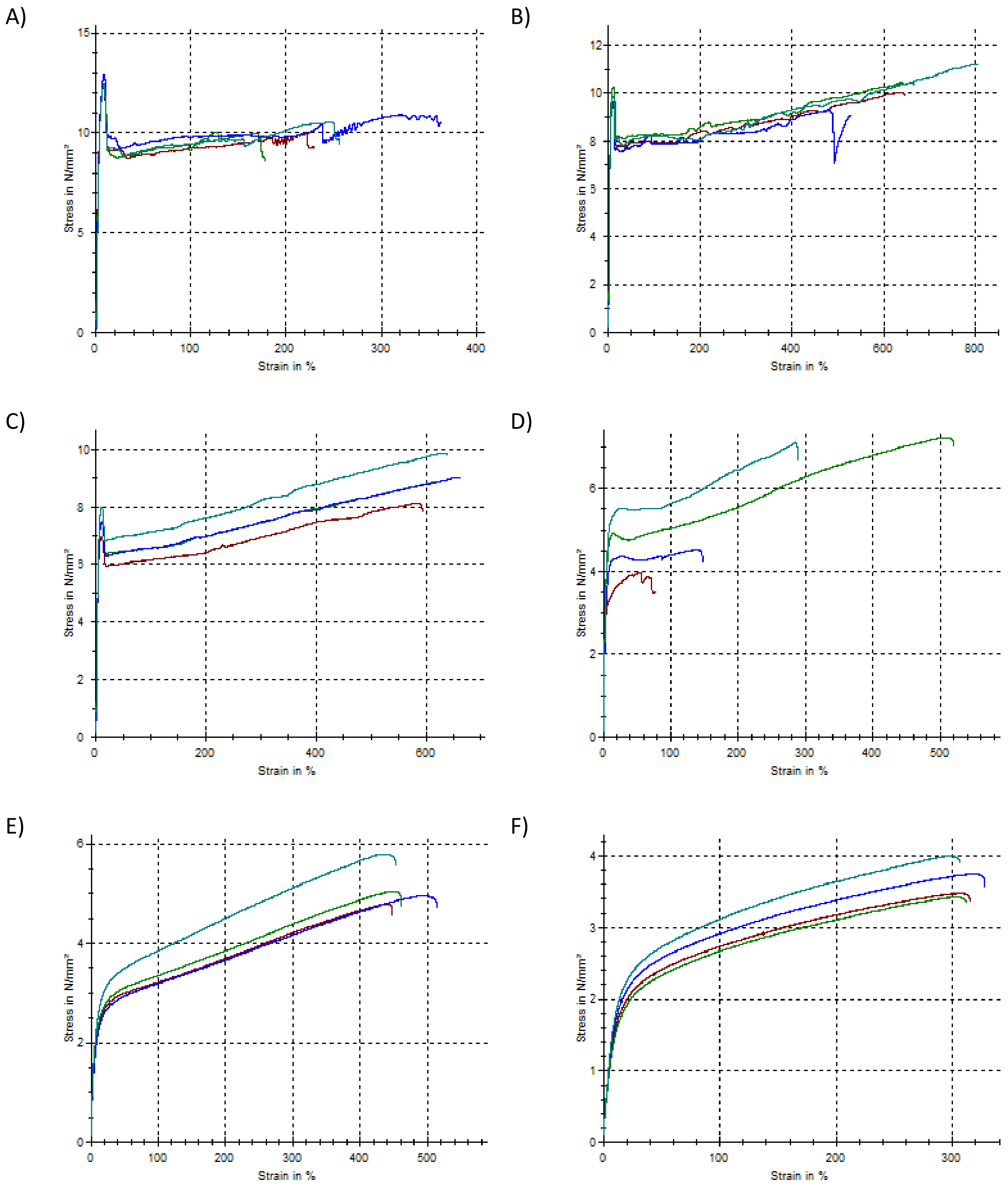

Figure S6. Example of the tensile curves of sample **P4** for A) pure **P4**, and **P4** blends with B) 4.8% IL C) 9.1% IL D) 16.7% IL E) 23.1% IL and F) 28.6% IL
